# Comparison of two chromogenic media and evaluation of two molecular based identification systems for *Enterobacter sakazakii *detection

**DOI:** 10.1186/1471-2180-6-15

**Published:** 2006-02-23

**Authors:** Angelika Lehner, Sabine Nitzsche, Pieter Breeuwer, Benjamin Diep, Karin Thelen, Roger Stephan

**Affiliations:** 1 Institute for Food Safety and Hygiene, Vetsuisse Faculty, University of Zurich, CH-8057 Zurich, Switzerland; 2 Nestle Research Center, Nestec Ltd, Ver-Ches-les-Blanc, Lausanne, Switzerland; 3 vermicon AG, 80992, Munich, Germany

## Abstract

**Background:**

*Enterobacter sakazakii *is a foodborne pathogen that has been associated with sporadic cases and outbreaks causing meningitis, necrotizing enterocolitis and sepsis especially in neonates. The current FDA detection method includes two enrichment steps, the subculturing of the second enrichment broth on a selective agar (VRBG), a further subculturing of selected grown colonies on TSA and the subsequent biochemical identification of yellow-pigmented colonies by API20E. However, there is a strong need for simplified methods for isolation and identification of *E. sakazakii*. In this study, two chromogenic media, which allow to indicate presumptive *E. sakazakii *colonies by the alpha glucosidase activity, as well as a newly developed 1,6-alpha-glucosidase based conventional PCR assay and a rRNA oligonucleotide probe based commercial test system for identification of presumptive *E. sakazakii *were evaluated on 98 target and non-target strains. The methods were compared with respect to specificity aspects.

**Results:**

A total of 75 presumptive *E. sakazakii *and 23 non-target strains were analysed by using chromogenic media, alpha-glucosidase based PCR assay, and the VIT assay. For most presumptive *E. sakazakii *strains on the chromogenic media, the PCR and VIT assay confirmed the identification. However, for a number of presumptive *E. sakazakii *isolates from fruit powder, the alpha-glucosidase PCR and VIT assay did not correspond to the typical *E. sakazakii *colonies on DFI and ESIA. Further characterization by API32E identification, phylogenetic analysis of partial 16S rRNA sequences and ribotyping strongly suggested, that these strains did not belong to the species *E. sakazakii*. The newly developed alpha-glucosidase based PCR assay as well as the commercially available VIT *Enterobacter sakazakii *identification test showed an excellent correlation with the 16S rRNA data, and are thus well suited for identification of *E. sakazakii*.

**Conclusion:**

The results indicate that presumptive colonies on ESIA and DFI media need further species identification. Both evaluated molecular methods, the alpha-glucosidase PCR and the 16S RNA in situ hybridisation test (VIT), although based on completely different target regions and methodologies performed equally well in terms of specificity.

## Background

*E. sakazakii *is a motile, non-spore forming, Gram negative facultative anaerobe microorganism. It was previously referred to as "yellow-pigmented *Enterobacter cloacae*" until it was designated a unique species by Farmer et al. [1].

The organism is an occasional contaminant of powdered infant formula milk that can cause a rare, but life threatening form of neonatal meningitis and necrotizing enterocolitis, which is the most common gastrointestinal emergency in the newborn [2,3].

The current Food and Drug Administration (FDA) method for detection of *E. sakazakii *includes a pre-enrichment procedure in buffered peptone water (BPW), enrichment in *Enterobacteriaceae *enrichment (EE) broth, plating on violet red bile glucose agar (VRBG) and picking of five grown colonies onto tryptone soy agar (TSA) plates, which are incubated at 25°C for 48–72 hours. Yellow-pigmented colonies, typical for *E. sakazakii*, on the TSA plates are confirmed using the API20E system. However, there is a strong need for simplified methods for isolation and identification of *E. sakazakii*. Guillaume et al. [4] published a new procedure, based on selective enrichment in modified lauryl sulfate tryptone broth, by addition of 0.5 M NaCl. Additionally, there is a strong need for good identification methods for presumptive *E. sakazakii *isolates. A feature, which can be used for this is the α-glucosidase activity, which was demonstrated to be present in all *E. sakazakii *strains and not in most other members of the *Enterobacteriaceae *[5]. Based on this biochemical property several differential media were developed recently [6-9]. Two of these media the Oxoid Chromogenic *Enterobacter sakazakii *Agar (Oxoid CM1055, Oxoid, UK) – also known as the Druggan-Forsythe-Iversen (DFI) formulation [7] – and *Enterobacter sakazakii *Isolation Agar (ESIA, AES, France), are commercially available and were included in our comparative study.

In a recent study, the molecular basis of the α-glucosidase activity in *E. sakazakii *was determined [10]. An open reading frame containing a sequence potentially coding for a 1,6-α-glucosidase was identified. In the current study, the potential of a PCR system based on the 1,6-α-glucosidase for the specific identification of *E. sakazakii *was evaluated on the DNA of target and non-target strains.

The VIT (vermicon identification technology) represents a commercially available detection and identification system based on fluorescently labelled gene probes targeting specified regions on the ribosomal RNA of the bacteria. Subsequent to analysis, the illuminated *E. sakazakii *cells are visualised under an epifluorescence microscope.

The current study represents a comparison of two commercial chromogenic media, which indicate presumptive *E. sakazakii *colonies, as well as an evaluation of an α-glucosidase based PCR assay and a rRNA targeting in situ technique for the rapid identification of *E. sakazakii*.

Ninety-eight target and non-target strains were included in the study and results were compared for specificity and convenience in performance.

## Results and discussion

### The chromogenic media

**Figure 1:**
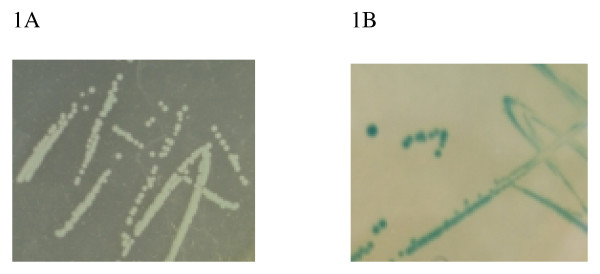
Atypical colonies of *E. sakazakii *FSM 322 (A) and typical colonies of *S. ficaria *(B) on DFI agar.

The results of the target and non-target strains included in this study for the comparison of the DFI and the ESIA media are summarized in table 1 and 2 in view of producing presumptive, typical colonies for *E. sakazakii*. Generally, the results for the 63 *E. sakazakii *strains were in good concordance, blue-green coloured colonies on DFI agar and turquoise on ESIA media, except for one *E. sakazakii *strain (FSM 322), which gave grey-white colonies on DFI agar (Figure 1A) and thus was considered negative on this medium. Furthermore, of the 23 non-target strains (Table 1), one *S. ficaria *environmental isolate showed the typical blue-green colour when grown on DFI agar, but was correctly identified as negative on ESIA medium (Figure 1B).

**Figure 2:**
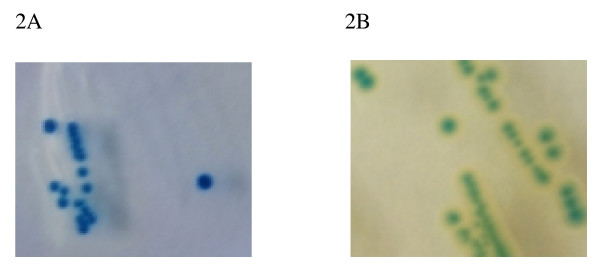
Non-*E. sakazakii *fruit powder isolate 1160/04 grown as typical colonies on ESIA (A) and DFI (B) media.

However, within a set of 12 strains, originally isolated from fruit powder (Table 2), typical blue green and turquoise colonies, respectively were observed for 8 strains on ESIA and for 11 isolates on DFI medium, when incubated at the recommended temperature. An example of the fruit powder isolate 1160/04 grown on DFI and ESIA medium is given in Figure 2A and 2B. API32E analysis on these strains revealed ambiguous results, but none of the strains were identified as *E. sakazakii*. According to the literature *P. shigelloides*, *E. vulneris*, *C. koseri *or *Pantoea *sp. can give typical coloured colonies on DFI agar [6]. API32E identification suggested as identification *E. vulneris *and *Pantoea *spp. as well as *Buttiauxiella agrestis [*11], which was previously known as *Citrobacter *group F, for several isolates within this group, although with low levels of confidence. However, the two reference strains *E. vulneris *ATCC 33821 and *P. agglomerans *ATCC 27155, included in this study were correctly identified as negative by both selective media (Table 1).

**Figure 3:**
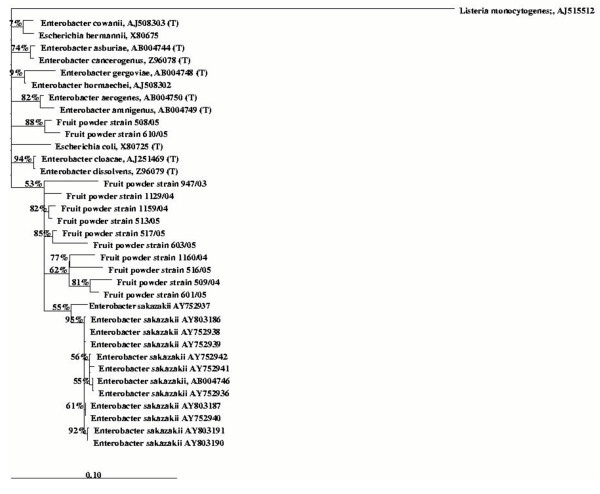
Phylogenetic tree comprising the partial 16S rRNA gene sequences of twelve non-*E. sakazakii *fruit powder isolates investigated in this study in comparison to a selection of previously sequenced *E. sakazakii *strains as well as other selected members of the genus *Enterobacteriaceae*.

**Figure 4:**
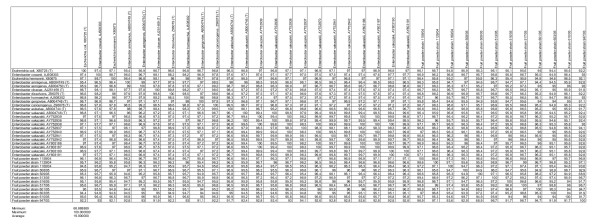
Respective distances matrix calculated with the sequences from figure 3.

In order to obtain more information on the fruit powder strains partial sequencing of the 16S rRNA gene was performed. Affiliation to the phylogenetic tree revealed, that all isolates were clearly distinct from *E. sakazakii *sequences from both lineages [12]. In Figure 3 and 4 the phylogenetic tree (Figure 3) and the respective distances matrix (Figure 4) of these strains with respect to *E. sakazakii *strains from both lineages and other selected members of the *Enterobacteriaceae *are given. Sequence similarities of 96 – 97% to type strains of other members of the *Enterobacteriacaeae *suggested that the organisms were members of the same genus but none of the isolates exhibited sequence similarities > 97% to *E. sakazakii *sequences from both lineages (Figure 4). Finally, ribotyping was performed on nine of the questionable strains again revealing, that the strains are not belonging to the *E. sakazakii *group (data not shown).

Additionally, growth experiments were performed for the strains of interest using a recently developed selective enrichment for *E. sakazakii *detection in environmental samples [4]. Experiments were performed in lauryl sulfate tryptose broth (mLST) supplemented with 0.5 M NaCl and 10 mg/liter vacomycin with or without an included non-selective enrichment step in BPW starting with BHI grown cultures. All strains were unable to grow within mLST broth at 44°C, regardless including a non-selective enrichment step in BPW or not (Table 2). In the study recently published by Guillaume-Gentil et al. [4], all of the *E. sakazakii *strains (n = 99) tested, were able to grow in mLST at 45°C, whereas 35 of 39 strains of potential competitors, all belonging to the *Enterobacteriaceae*, were suppressed. They concluded, that the inclusion of this enrichment broth could be very useful for the reliable detection of *E. sakazakii *in environmental samples. In our study, however, *E. sakazakii *strain FSM 322 could also not grow in mLST.

The α-glucosidase based PCR

**Figure 5:**
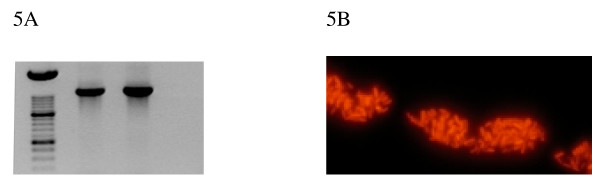
(A): Strain 1160/04 identified as *E. sakazakii *by the α-glucosidase based PCR assay (A); lane 1: DNA 100 bp marker, lane 2: positive control (*E. sakazakii *reference strain ATCC 29004), lane 3: *E. sakazakii *fruit powder isolate FSM 322, lane 4: negative control. (B) Positive VIT result of strain FSM 322

Applying the α-glucosidase based PCR system we were able to identify all target strains. No false positive result was obtained within the group of non-target strains. An example of the identification of a fruit powder strain by PCR assay is given in Figure 5A.

It is worth mentioning that both compared selective media are based on the proposed α-glucosidase activity of *E. sakazakii*. From literature it is known, that several other organisms also exhibit this biochemical feature and can grown on DFI [7]. The α-glucosidase based PCR system, however, exclusively targets the gene responsible for the α-glucosidase activity in *E. sakazakii*.

VIT (vermicon identification technology)

By using the VIT test, all of the *E. sakazakii *strains were identified. No false positive result was obtained within the group of non-target strains. These results were in good concordance with those obtained with the α-glucosidase based PCR system. The target strains were easy to identify due to the specific red colour under the epifluorescence microscope. In Figure 5B, an example of a positive identification of *E. sakazakii *fruit powder strain1160/04 by VIT is given. However, during analysis of the strains used in the study, some autofluorescence was observed in several samples of the non-target strains. Inclusion of a positive reference strain during analysis can overcome this problem, since a "true signal" differs significantly in brightness.

The major advantage of this easy to handle molecular method is represented by the fact, that the bacterial rRNA is targeted by the probes, thus in principle only vital *E. sakazakii *cells containing an efficiently high ribosomal content are detected by the assay. Results are obtained within three hours and the performance of the test is not restricted to an especially equipped laboratory.

Detection is possible to the single cell level, even in mixed material containing target and non-target cells. The detection limit of the assay was experimentally determined using serial dilutions of overnight cultures of two different *E. sakazakii *reference strains (ATCC29544, ATCC51329) and determination of the cfu/ml by quantitative plating. A detection limit of 10^3 ^cfu/ml was ascertained for both strains.

## Conclusion

By comparing the two chromogenic media on a set of 98 target and non-target strains, the ESIA medium proved to be more indicative for presumptive *E. sakazakii *colonies. Inclusion of an enrichment step in mLST can reduce the number of presumptive colonies, with are not *E. sakazakii*. Nevertheless, all presumptive colonies on ESIA and DFI media need a further species identification. The two molecular methods, although based on completely different target regions and methodologies performed equally well in identifying *E. sakazakii *strains. Both methods showed 100% specificity. The PCR based system needs a DNA extraction step, prior to amplification set up, but is easily implemented into PCR adapted laboratories. The VIT test represented a fast and convenient to handle test system.

## Methods

Target strains

63 *E. sakazakii *strains from human, food, cosmetics and environmental origin including three reference strains were used in the study (Table 1).

Non-target strains

A first set of 39 strains belonging to different species other than *E. sakazakii *represented a part of the non-target group (Table 1).

Moreover, another 12 strains were added to this non-target group (table 2). They were isolated from fruit powder using the FDA approach. All of these strains have shown typical colonies on ESIA and/or DFI agar and were shown by API32E to be non-*E. sakazakii*. However, it was not possible to reliable identify theses strains by API32E. In order to do this, 16S rRNA gene sequencing and phylogenetic analysis of these fruit powder strains was done. For PCR amplification of the 16S rRNA gene, DNA was extracted from 1 ml cultures grown in BHI broth for 24 hours at 37°C using the DNeasy Tissue Kit (Qiagen AG, Switzerland) in accordance with the protocol of the supplier. Partial sequencing of the 16S rRNA genes of these strains was performed according to the method recently published by Lehner et al. [12]. The same primers were used for amplification and sequencing of the 16S rRNA gene, except that no internal walking primers were employed. The partial 16S rRNA gene sequences of the 12 strains were added to an alignment of 28.000 almost full length small subunit rRNA sequences by using the alignment tool of the ARB program package. Phylogenetic analyses were performed using distance matrix and the TREEPUZZLE tool included in the ARB software package employing special data structures (PT-servers) derived from the ssu-rRNA database „ssu_jan04.arb.

Additionally, ribotyping was performed on nine selected fruit powder strains (1159/04, 947/03, 1129/04, 517/05, 610/05, 1160/04, 513/05, 509/05, 508/05) on a DuPont RiboPrinter Microbial Characterization System^*TM *^according to the method described by Bruce [13].

Furthermore, growth of the fruit powder strains in lauryl sulfate tryprose broth supplemented with 0.5 M NaCl and 10 mg / liter vancomycin (mLST) for 22 – 24 h at 44 ± 0.5°C [4] was examined in two different manners: i) inoculating 10 ml of mLST broth with 10 – 100 cfu of a BHI grown culture (overnight, 37°C), ii) inoculating 25 ml of buffered peptone water (BPW) (37°C for 16 – 20 h) with 10 – 100 cfu of a BHI grown culture (overnight, 37°C). Thereafter 10 ml of the mLST broth were inoculated with 0.1 ml of the BPW grown culture and incubated as described above.

### Chromogenic media

The strains were streaked onto DFI agar plates (Oxoid, UK) and ESIA agar plates (AES, France) and incubated at 37°C (DFI) and 44°C (ESIA) for 24 h. Colonies that were entirely blue-green on DFI and turquoise on ESIA media after 24 h incubation were indicative for presumptive *E. sakazakii *colonies. According to the suppliers protocol purple colonies on ESIA media and non-blue green colonies on DFI agar were considered non typical.

### Alpha glucosidase specific PCR

Within a recent study two open reading frames were identified both coding for enzymes with the potential to hydrolyze the fluorescent substrate 4-methylumbellyferryl-α-D-glucoside [10]. The primers that were used for subcloning in that study were modified in the current study and the optimal annealing temperature was determined. The following primers were applied for specific amplification of the gene putatively encoding the α-glucosidase activity: EsAgf: 5'- TGA AAG CAA TCG ACA AGA AG-3'and EsAgr: 5'- ACT CAT TAC CCC TCC TGA TG-3' generating a product of 1680 bp in size (GenBank accession number AM075208). The PCR reactions were set up in a total volume of 50 μl. The reaction mixture contained 5 pmol primers each, 100 μM dNTPs each, 1× *Taq *polymerase buffer and 2 U *Taq *polymerase (Promega). Thermal cycling was carried out by using an initial denaturation step at 94°C for 2 min, followed by 29 cycles of denaturation at 94°C for 30 sec, annealing at 58 °C for 60 sec and extension at 72°C for 90 sec. Cycling was completed by a final elongation step at 72°C for 5 min. The amplification products were analysed by gel electrophoresis and ethidium bromide staining.

### Vermicon identification technology (VIT)

The VIT *Enterobacter sakazakii *test (vermicon, Munich, Germany) was performed according to the instructions of the manufacturer. For analysis, 1 ml of BHI grown cultures (overnight, 37°C) were used.

Accession numbers of 16S rRNA genes of fruit powder strains included in the study:

Strain 610/05 (GenBank: DQ273680), strain 508/05 (GenBank: DQ273681), strain 516/05 (GenBank: DQ273682), strain 1159/04 (GenBank: DQ273683), strain 601/05 (GenBank: DQ273684), strain 603/05 (GenBank: DQ273685), strain 1160/04 (GenBank: DQ273686), strain 1129/04 (GenBank: DQ273687), strain 513/05 (GenBank: DQ273688), strain 517/05 (GenBank: 273689), strain 509/05 (GenBank: DQ273690), strain 947/03 (GenBank: DQ273691)

## Authors' contributions

AL carried out the PCR experiments, the phylogenetic analyses and drafted the manuscript. SN participated in the PCR experiments and carried out the cultural and VIT experiments. PB and BD performed ribotyping and participated in the cultural experiments. KT carried out the 16S rRNA gene sequencing. RS conceived of the study, participated in its design and coordination and helped to draft the manuscript. All authors read and approved the final manuscript.

## Figures and Tables

**Table 1:** Target and non-target strains used in this study and results obtained with the chromogenic media DFI and ESIA, the α-glucosidase based PCR assay and the rRNA probe based VIT test.

Species (number of strains)	Origin	ESIA*	DFI^+^	PCR	VIT
*E. cloacae *(1)	ATCC13047	neg	neg	neg	neg
*E. cloacae *(1)	LMG3008	neg	neg	neg	neg
*E. cloacae *(6)	clinical, cosmetics	neg	neg	neg	neg
*E. cloacae *(2)	food	neg	neg	neg	neg
*E. dissolvens *(1)	ATCC23373	neg	neg	neg	neg
*E. hermanii *(1)	wild strain	neg	neg	neg	neg
*P. agglomerans *(1)	ATCC27155	neg	neg	neg	neg
*P. agglomerans *(2)	food, cosmetics	neg	neg	neg	neg
*Pantoea sp*. (1)	food	neg	neg	neg	neg
*K. oxytoca *(1)	cheese	neg	neg	neg	neg
*S. liquefaciens *(1)	cosmetics	neg	neg	neg	neg
*S. ficaria *(1)	production environment	neg	**pos**	neg	neg
*Serratia sp*. (1)	cosmetics	neg	neg	neg	neg
*Salmonella *Enterititis (1)	wild strain	neg	neg	neg	neg
*S. sonnei *(1)	wild strain	neg	neg	neg	neg
*E. coli *(1)	ATCC25922	neg	neg	neg	neg
					
*E. sakazakii *(1)	ATCC29544	pos	pos	pos	pos
*E. sakazakii *(1)	ATCC29004	pos	pos	pos	pos
*E. sakazakii *(1)	ATCC51329	pos	pos	pos	pos
*E. sakazakii *(20)	fruit powder^1^	pos	pos	pos	pos
*E. sakazakii *(5)	cosmetics	pos	pos	pos	pos
*E. sakazakii *(15)	human^2^	pos	pos	pos	pos
*E. sakazakii *(1)	milk powder^3^	pos	pos	pos	pos
*E. sakazakii *(2)	baby food ^4^	pos	pos	pos	pos
*E. sakazakii *(1)	milk ^5^	pos	pos	pos	pos
*E. sakazakii *(15)	production environment^6^	pos	pos	pos	pos
*E. sakazakii *(1)	production environment	pos	**neg**	pos	pos

* positive = turquoise colonies typical for *E. sakazakii *

^+ ^positive = blue-green coloured colonies typical for *E. sakazakii *

^1^including strains (Acc. number): 858 (AY752936), 759 (AY752939), 954 (AY752938), AY752943

^2^including strains (Acc. number): ES 11 (AY803187), ES 4 (AY803186)

^3^including strain (Acc. number): FSM 265 (AY803191)

^4^including strain (Acc. number): FSM 393 (AY752941)

^5^including strain (Acc. number): FSM 33 (AY752940)

^6^including strains (Acc. number): FSM 468 (AY752942), FSM 266 (AY803190)

**Table 2:** Colonies on ESIA and DFI media, growth in mLST broth and results of the two molecular identification methods for the 12 non-*E.sakazakii *fruit powder isolates investigated in this study.

Fruit powder strain	colonies on ESIA*	colonies on DFI^+^	growth in mLST at 44°C	α-glucosidase PCR	VIT test
947/03	non typical	typical	no growth	neg	neg
1129/04	typical	typical	no growth	neg	neg
1159/04	typical	typical	no growth	neg	neg
1160/04	typical	typical	no growth	neg	neg
508/05	non typical	typical	no growth	neg	neg
509/05	typical	typical	no growth	neg	neg
513/05	typical	typical	no growth	neg	neg
516/05	typical	non typical	no growth	neg	neg
517/05	typical	typical	no growth	neg	neg
601/05	typical	typical	no growth	neg	neg
603/05	non typical	typical	no growth	neg	neg
610/05	non typical	typical	no growth	neg	neg

* typical = turquoise colonies typical for *E. sakazakii *

^+ ^typical = blue-green coloured colonies typical for *E. sakazakii*
